# MultiOptForest: An interactive multi-objective optimization tool for forest planning and scenario analysis

**DOI:** 10.12688/openreseurope.15812.2

**Published:** 2024-12-16

**Authors:** Kyle Eyvindson, Daniel Burgas, Clara Antón-Fernández, Jussi Hakanen, Michael Emmerich, Julian Klein, Mikko Mönkkönen, Tord Snäll, Astor Toraño Caicoya, Marta Vergarechea, Clemens Blattert

**Affiliations:** 1 Faculty of Environmental Sciences and Natural Resource Management, Norges miljø- og biovitenskapelige universitet, Ås, NO-1432, Norway; 2 Natural Resource Institute Finland (LUKE), Laatokartanonkaari 9, Helsinki, 00790, Finland; 3 Department of Biological and Environmental Science, Jyvaskylan yliopisto, P.O. Box 35, Jyväskylä, 40014, Finland; 4 School of Resource Wisdom, Jyvaskylan yliopisto, P.O. Box 35, Jyväskylä, 40014, Finland; 5 Division of Forest and Forest Resources, Norwegian Institute for Bioeconomy Research, Ås, 1433, Norway; 6 Silo Ai, Fredrikinkatu 57 C, Helsinki, 00180, Finland; 7 Leiden Institute of Advanced Computer Science, Universiteit Leiden, Leiden, South Holland, 2333CA, The Netherlands; 8 Swedish Species Information Centre, Sveriges lantbruksuniversitet, P.O. 7007, Uppsala, 75007, Sweden; 9 Chair of Growth and Yield Science, School of Life Sciences, Technische Universitat Munchen, Hans-Carl-von-Carlowitz-Platz 2, 8354 Freising, Germany; 10 Forest Resources and Management, Swiss Federal Research Institute WSL, Birmensdorf, 8903, Switzerland

**Keywords:** Multi-objective optimization, forest planning, scenario assessment, interactive decision making

## Abstract

MultiOptForest is an open-source software designed to simplify building and solving multi-objective optimization problems for forest planning. It aims to find the optimal portfolio of management regimes that balance the objectives regarding multiple forest ecosystem services and biodiversity. The software flexibly imports data, allowing for the use of a variety of forest simulator outputs. The user provides preference information through a user-friendly graphical interface, where the range of possible values for each objective is provided. MultiOptForest solves the optimization problem producing a set of Pareto optimal solutions, *i.e.,* solutions where none of the objectives can be improved without compromising others. MultiOptForest is versatile enough to design a Pareto optimal forest plan for a small holding to assess management and the trade-off between multiple policy objectives impacting the future development of forests across regions and countries.

## Introduction

Forests play a critical role in providing multiple ecosystem services and biodiversity, which highlights the importance of planning the use of forests at all levels, from small-scale private forest holdings ( Pohjanmies *et al.*, 2017) to national or international policy evaluations ( Mazziotta *et al.*, 2022). This has led to a rising demand for tools to quantify the conflicts between multiple forest objectives. These tools should be able to investigate the trade-offs and synergies between objectives, in order to plan the silvicultural and harvesting decisions to be taken to meet a wide range of preferences ( Linkevičius *et al.*, 2019; Nordström *et al.*, 2019). To meet a wide range of use cases, a general optimization tool needs to be (a) intuitive to use, to allow for a broad range of users with limited computer skills; (b) powerful and able to handle a large variety of problems from forest holdings to national or even international scales; (c) flexible, *i.e.,* can accommodate disparate types of forest growth information provided by different forest simulators; (d) can account for the several categories of objectives that are expected from forests; and (e) transparent, to facilitate evaluation of the delivered results.

With these motivations in mind, we created the MultiOptForest software ( Eyvindson *et al.*, 2023), which utilizes a structured optimization process with a strong theoretical foundation. It assists the human decision maker to analyse the huge search space of possible management regimes for preferable Pareto optimal solutions ( Borges *et al.*, 2014; Díaz-Yáñez *et al.*, 2021). These are solutions that can no longer be improved in one objective without deteriorating other objectives ( Miettinen, 1999). Thereby win-win strategies are always favored whereas all lose-lose strategies are excluded, and in case of trade-off the user can adjust her/his preferences in an interactive dialogue with the software. 

The MultiOptForest software builds upon the history of multi-objective optimization frameworks used to create optimized management plans in forestry applications. A few decades ago, forest planning literature often focused on the production of timber resources, aiming for an optimal even-flow of timber over time while minimizing costs of harvesting and logistics ( Johnson & Scheurman, 1977). The underlying optimization problems have often been highly structured, focusing on harvest intensity and economic priorities. Nowadays, forests are recognized for their contribution to a wide range of societal demands ( Blattert *et al.*, 2022; Winkel *et al.*, 2022; Pascual, *et al.*, 2017; MEA 2005), and this is why there is a need to shift towards a flexible approach accounting for those multi-objectives (e.g. Augustynczik *et al.*, 2020; Eggers *et al.*, 2022; Uhde *et al.*, 2017). The interactive optimization approach used in MultiOptForest is based on optimizing an achievement scalarizing function to find the multi-objective solution closest to the preference information ( *e.g.* demands for ecosystem services) specified by the user. The methodology is what is presented in the desdeo-mcdm package (RRID:SCR_023502) in the DESDEO framework ( Misitano *et al.*, 2021), however our software is adapted to forest management planning problems, data and optimization. The MultiOptForest software is also related to the optimLanduse software ( Husmann *et al.*, 2022), which optimizes the spatial land use cover compositions to provide a range of ecosystem functions, biodiversity indicators and social preferences. However, optimLanduse uses an robust multi-objective land-cover composition optimization approach of Knoke *et al.* (2016), while MultiOptForest used a multi-objective problem formulation ( Miettinen, 1999) in combination with the achievement scalarizing functions approach ( Wierzbicki, 1986). 

MultiOptForest allows advanced users to connect forest simulator data to an interactive optimization framework that non-expert users ( *e.g.*, policy makers, consultants, forest owners, non-governmental organizations) can adjust according to their preferences in an intuitive and transparent manner, without being distracted by the details of the algorithm design and parameters. The MultiOptForest software is designed to use open-source solutions for optimization, although the option to use commercial optimization solvers remains available. The software provides a straightforward approach to construct optimization problem formulations and a systematic method for eliciting preferences from non-expert users.

The input requirements of this software are projections of multiple alternative management trajectories or silvicultural treatments for each forest stand or plot under consideration. This is provided through forest simulation software ( Antón-Fernández & Astrup, 2022; Pretzsch *et al.*, 2008; Pretzsch *et al.*, 2002; Rasinmäki *et al.*, 2009; Wikström *et al.*, 2011), where the output represents alternative potential stand-level scenarios dependent on the decisions taken in the forest. The user can define the individual optimization objectives based on the simulator outputs, for instance as an indicator for biodiversity the user may strive to maximize deadwood at a specific year. Additionally, the objective functions should be based on the interest and ability of the forest owner to provide preference information. Determining appropriate optimization objectives currently requires an advanced user, someone who understands both the simulated forest data and the appropriate interpretations of the parameters used to set the optimization objectives. Ongoing development of the software will integrate the construction of objectives with the graphical user interface, allowing more flexibility and usability of the software. Once the objectives are determined and the multi-objective problem has been formulated, the user can interactively provide preference information and explore the corresponding Pareto optimal solutions to better understand the range of Pareto optimal management scenarios and conflicts or trade-off between objectives.

The MultiOptForest software has so far been used to assess and quantify the coherence and incoherence of forest-oriented policies across Fennoscandia ( Blattert *et al.*, 2022; Vergarechea *et al.*, 2023) and in Germany ( Toraño Caicoya *et al.*, 2023). These studies indicate how to translate forest-related policies into comprehensive optimization problems and show how the identified forest management scenario can lead to meeting the objectives of the policies. As a flexible approach to constructing multi-objective optimization formulations, this software can be used in a variety of forest planning cases, including large scale strategic planning cases (e.g. national forest inventory plot level) as well as small scale tactical and operational planning cases (e.g. individual forest stands of a management unit) ( Kangas *et al.*, 2015).

## Software description

This optimization framework has been constructed entirely in Python (RRID:SCR_008394), using a variety of open-sourced packages: Pandas, NumPy, MatPlotLib (RRID:SCR_018214, RRD:SCR_008633, RRID:SCR_008624). The backbone package for the optimization is OR-Tools ( Perron & Furnon, 2022), which we use to construct the optimization problem. This approach allows for the use of openly available solvers (such as coin-or branch and cut (CBC), coin-or linear programming (CLP), Google linear optimization package (GLOP)) and commercial solvers (such as CPLEX, GUROBI ^TM^, XPRESS). For small problems, open solvers are quite capable, but larger problems may require the use of commercial solvers and may require more computational power to conduct the optimizations in a reasonable timeframe. The specific minimum system requirement depends on the size of the input data. However, the example cases found in the source code ( Eyvindson *et al.*, 2023, or from Code Ocean) were able to be run on a virtual machine running Ubuntu 18.04, with 3 Gb of ram.

### Software architecture

**Figure 1.**
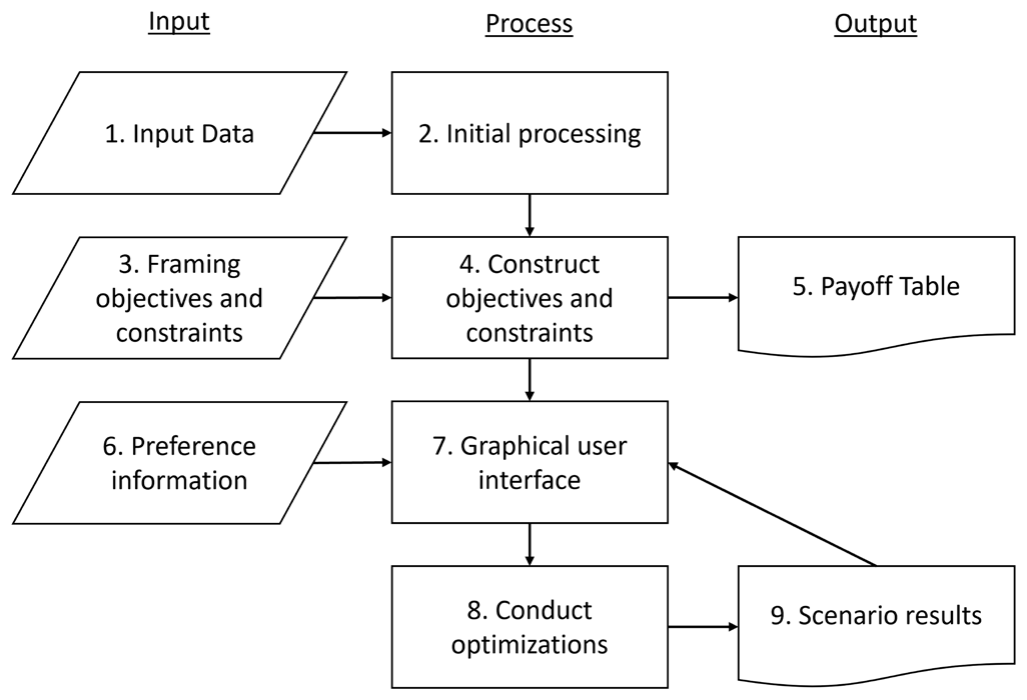
The architecture of the software. The user can input data [component 1], design objectives and constraints [component 3] and provide preference information [component 6]. The model construction, processing of data and output of solutions is automated.

***Individual components.*** The architecture of the software is composed of nine components ( Figure 1). The first component focuses on importing simulated forest data. This component converts the data provided to useful format for optimization. The software currently imports data as a large table based on specific structural principles related to forest management options (e.g. effect on ecosystem services and biodiversity). To allow for different forest simulators, this component can be edited to allow for more diverse data types. Data is stored as a table with an index for each forest stand or plot, each forest management option and for each simulated year.

The second component processes the data and constructs the model base for the optimization problem. A framework for the optimization problem is constructed, facilitating the addition of introduction of objectives and constraints. The third component allows for (advanced) users to construct problem specific objectives and constraints for the problem. How this is performed is described further in the sections “Formulating objective functions and epsilon constraints” and “Formulating additional constraints.” The fourth component integrates these objectives and constraints into the multi-objective optimization model, following the structure as described in “Multi-objective problem” section. To construct the objectives, the software solves a series of maximization problems for each objective function. This results in a payoff table (component 5) presenting the ideal and nadir values for each objective, which is used to normalize the objectives and to provide users a reference when providing preference data.

The remaining components interact with preference information provided by the user. Components six and seven relate to the user to quantify their preferences, with component seven being the graphical user interface (GUI) where the user inputs the preference information. When the user inputs preferences they can interact and exploring specific solutions generated by the multi-objective optimization problem through various visualization techniques ( Miettinen, 2014). Preference information (component six) is provided as target values (objectives) or minimum threshold values (constraints). Components eight and nine focus on optimizing specific issues based on the preference information from the decision-maker and quantifying the solution for reintroduction into the GUI in component seven. This enables the extraction of solutions that align with the decision-maker's preferences and decision outputs for each forest stand or plot, ensuring a tailored approach to forest management optimization.

**Figure 2.**
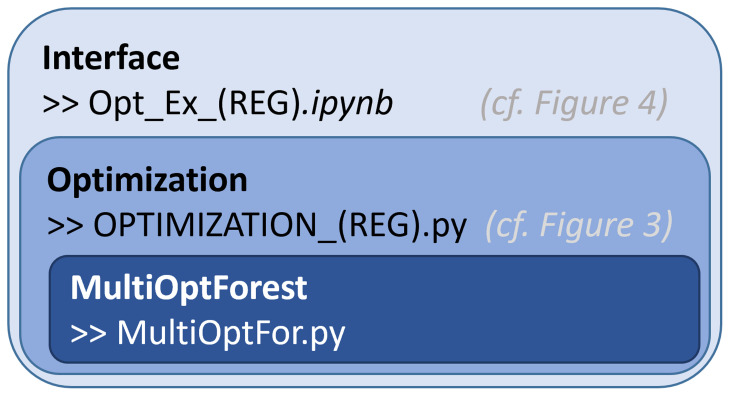
Different levels of the software organized based on complexity of the code. Interface – users can interact with (allows for setting of objectives and constraints). Optimization – code that links the core functions to prepare for the interactive component. MultiOptForest – basic functionality used to conduct the optimization and visualization. REG refers to the regional specific code allowing for minor adaptions to input data and objectives.

***Levels of software organization.*** To enable ease of interaction with the multi-objective optimization component, we constructed a three-layer organization of the code ( Figure 2). At the top level, we have the user interface, where the user can provide only the core information required; the forest simulator data and the preferences on ecosystem services. At this level, the user can identify the location of the data and provide preference information for the objectives selected in the multi-objective optimization problem. These objectives are predefined, set by an advanced user in the middle layer of the code.

The second layer of the code allows for more generalizability of the functionalities defined in the third layer of the code. This second layer contains forest simulator-specific information, allowing for flexible interpretation of data, and allows more advanced users to pre-construct objective functions. This will require the user to have a comprehensive understanding of the forest simulation data and the competence to construct python dictionaries according to a predefined template. This code calls specific functions to construct the multi-objective optimization problems in an organized fashion.

The third layer of the code is the core of the software, which is used to construct an object containing the core functions used in optimization and visualization. As the heart of the code, it is designed to flexibly add objective functions, constraints and calculate the key information to construct the multi-objective optimization problem.

Our software has been tested to import data from four forest simulators (SIMO, SiTree, Silva, Heureka), and minor modifications to the second layer of the software will be required to ensure consistency of the data and its interpretation.

### Software functionalities

***Importing data.*** Data can be imported from various forest simulators, we have adapted the software to import data from SIMO ( Rasinmäki *et al.*, 2009), SiTree ( Antón-Fernández & Astrup, 2022), Silva ( Pretzsch *et al.*, 2008; Pretzsch *et al.*, 2002), and Heureka ( Wikström *et al.*, 2011). MultiOptForest required only minor modifications of the second layer to ensure consistency of the data and its interpretation. These forest simulators provide projections of the development of the forest across time. The projections depend on the forest management regime implemented, which can include the specific timing or intensity of operations (thinning, final felling). The simulations can be at the forest stand level (a relatively homogeneous parcel of forest land) or the forest plot level (a fixed size of forested land, obtained using sampling approaches to represent larger forest areas).

Data can be provided from a variety of different forest simulators, but a minimum standard set of information from all forest simulators is required, indexed data (stand ID, year, management regime) and columns for periodical indicators representing the state of the forest and of the ecosystem services and biodiversity (country or model specific). Those indicators are simulated for each forest entity (forest stand or plot level) under alternative managements by the forest simulators.

Examples for the importing data of the above four forest models are on Code Ocean and can be interactively loaded for the below use case following the step-by-step guideline in the supplementary material. 

***Formulating objective functions and epsilon constraints.*** The procedure to create the individual objective functions and epsilon constraints is based on a pre-defined set of options ( Listing 1). For any of the forest simulator outputs, it is possible to formulate objectives and epsilon constraints related to temporal development of the specific output. In the current version of the code, there are 12 options for how the temporal aspect is treated (for a list of all, Table 1). For instance, from an ecological perspective, to meet biodiversity targets, we may want to increase the quantity of deadwood in the forest by a specific year, and to ensure that quantity is kept for the rest of the planning horizon. From a timber harvesting perspective, the objective could be to maximize the minimum or to ensure a minimum yearly increase in harvests across the planning horizon. The key differentiation between an objective function, and an epsilon constraint is that a target value is used to identify the preference of an objective, while a threshold value (where anything below is unacceptable) is used for an epsilon constraint). The implementation of each objective is based on eight attributes, linking the data to a specific temporal (c.f. Table 1) and spatial aggregation interpretation, see Listing 1 for examples. Based on these attributes the more advanced user is able to build individual objective functions depending on the optimization targets.

**Table 1.** Advanced users can handle temporal aspects in MultiOptForest when constructing individual objective functions. Each coded interpretation specifies how to implement these temporal aspects when solving the optimization problem.

Function Abbreviation	Plan language interpretation of the temporal aspect
min	Maximize the minimum value over the time horizon
average	Maximize the average value over the time horizon
firstYear	Maximizes the value of the first year
sum	Maximizes the sum of the values over the time horizon
targetYear	Aims to reach a specific target value at a specific year
targetYearWithSlope	Aims to reach a specific target value at a specific year, with a continued linear increase afterwards
lastYear	Maximize the value of the last year
periodicTargets	Meet a specific target value for all years
minYearlyIncrease	Minimize the yearly increase across the time horizon
maxYearlyIncrease	Maximize the yearly increase across the time horizon
minDecreaseDuringNPeriods	Minimize the periodic decrease across the time horizon
maxIncreaseDuringNPeriods	Maximize the periodic increase across the time horizon


1 OBJ.biodiversity = {
2   #Deadwood – target 2050, increase by XX%
3   "relative_Amount_Deadwood_2050":["Total Deadwood volume by 2050 (%, relative to 2016 values)", 
4                                       "Relative_Total_V_total_deadwood",
5                                       "max","targetYearWithSlope","sum", 2050]}
6 OBJ.wood_production = {
7   #Harvested roundwood – target 2025
8   "Total_Harvested_V_2025":["Total annual harvested timber volume by 2025 (log&pulp) (m3)", 
9                                          "Total_Harvested_V",
10                                         "max","targetYearWithSlope","sum", 2025]}


Listing 1: Two examples defining objective functions relating to forest biodiversity and wood production. The dictionary key is a unique description of the objective (e.g. Total_Harvest_V_2025), and the value of the dictionary is a 6-element list. The elements in the lists starting at lines 3 and 8 are 1) a human-readable string, 2) the column name of the simulation output dataset, 3) “max” or “min” if increase or decrease is aimed for, 4) how to handle temporal aspects, 5) how to handle spatial aspects, 6) the target year or a string of periodic targets (may not be required depending on how the temporal aspects are handled in 4). (cf. Blattert *et al*., 2022).

***Formulating additional constraints.*** The majority of the components should be formulated as optimization formulations and epsilon constraints, however there are situations when optimizing an aspect is not needed but only restrictions are required. To facilitate this, our program can generate two types of constraints that can 1) restrict management options on specified stands or plots ( *e.g.*, protected areas cannot be harvested), 2) require indicators not to exceed a specific reduction ( *e.g.*, to avoid species extinction). For example, the first constraint type can be used to limit management on drained peatlands to be only managed using either no management, or continuous cover forestry ( Listing 2). The second constraint type can be used to ensure specific threatened species not to go below a specific threshold (see documentation on the git).



1 OBJ.mfo.defineObjectives(OBJ.objectives,initialValues=OBJ.initialValues)
2 OBJ.CCFregimes = [regime for regime in OBJ.mfo.regimes if "CCF" in regime] + ["SA"]
3 OBJ.constraintTypes = {"CCFonPeat": 
4          ["Allowed regimes", "Only CCF on peat lands", OBJ.CCFregimes, "PEAT"]}
5 OBJ.mfo.defineConstraint(OBJ.constraintTypes)



Listing 2: Example of an enabled constraint that guarantees that only certain managements (different variants of continuous cover forestry [CCF] and set aside [SA]) are allowed on forest stands situated on peatland (“PEAT”). The dictionary key is a unique description of the constraint, and the value of the dictionary is a 4 or 5-element list, depending on the constraint type. For the “Allowed regimes” constraint type: The elements on line 4 are 1) the constraint type, 2) a human-readable string, 3) the management regimes allowed and 4) the column that identifies the stands or plots where the restriction occurs. For other constraint types, the variables of the list will vary slightly.

***Multi-objective problem.*** The core component of the software is where the optimization problem is constructed. To ease the implementation, we use a theoretically sound multi-objective problem formulation to find efficient solutions for each optimization scenario. This is accomplished using the core of multi-objective optimization ( Miettinen, 1999):


$$\begin{array}{c} \underset{x}{\text{minimize}}\;\{f_{1}(x),\ldots ,\;f_{n}(x)\}\\ subject\;x\in S \end{array}\;(1)$$

where Equation 1 describes an optimization that aims to simultaneously minimize a set of *n* objective functions (or simply ‘objectives’) *f _i_* ( *x*), *i* = 1, ..., *n* , with *x* being the decision vector for the management regimes, and *S* is the decision space, *i.e.,* the set all feasible management regimes. In this formulation, all the objectives are to be minimized. If some objective is to be maximized, it is equivalent to minimize – *f _i_*.

The technical implementation of the optimization was accomplished using two components. The first component uses the achievement scalarizing function of Wierzbicki (1986). This component uses reference points to quantify the preferences between the defined objectives. The second component uses the ε-constraint method ( Miettinen, 1999), which sets strict requirements for the specific objective and can be interpreted as a maximal (or minimal) level for each objective. Our software will construct both the achievement scalarizing function and epsilon constraint for each objective defined in the ‘formulating objective functions’ section.

The generalized formulation of the combined multi-objective problem formulation is a combination of the achievement scalarizing function to be minimized ( Hartikainen *et al.*, 2016), while incorporating the ε-constraint method:


$$\begin{array}{c} \underset{x}{\min}\underset{1\leq i\leq n}{\max}\left(f_{i}(x)-z_{i}^{ref}\right)/\left(z_{i}^{ideal}-z_{i}^{nadir}\right)\\ +\rho \sum_{i=1}^{n}f_{i}(x)/\left(z_{i}^{ideal}-z_{i}^{nadir}\right) \end{array}\;(2)$$

subject to:


$$\begin{array}{c} f_{i}(x)\leq \varepsilon_{i},\;i=1,\ldots ,n\\ x\in S \end{array}\;(3)$$

Equation 2 evaluates the distance away from ideal vector *z ^ideal^* ∈ *R ^n^* consists of the optimal values for each objective when optimized individually while the nadir vector *z ^nadir^* ∈ *R ^n^* consists of the worst values for each objective. The reference point *z ^ref^* ∈ *R ^n^* is constructed by using the user-defined aspiration levels, the ideal vector and the nadir vector depending on the preferences for the objectives. The second half of the equation is an augmentation term that guarantees that the solutions are Pareto optimal, with *ρ* set as an arbitrary small positive constant (the choice of the value may depend on the software, solver or other technical choices). Equation 3 is the ε-constraint so that each objective meets or exceeds the value from vector ε. An interesting technical detail is that the achievement scalarization function is not using the Euclidean distance to the reference point, but the Chebychev distance to the reference point. This choice leads to a more balanced approximation of the aspiration levels for the different objectives.

### Graphical user interface

**Figure 3.**
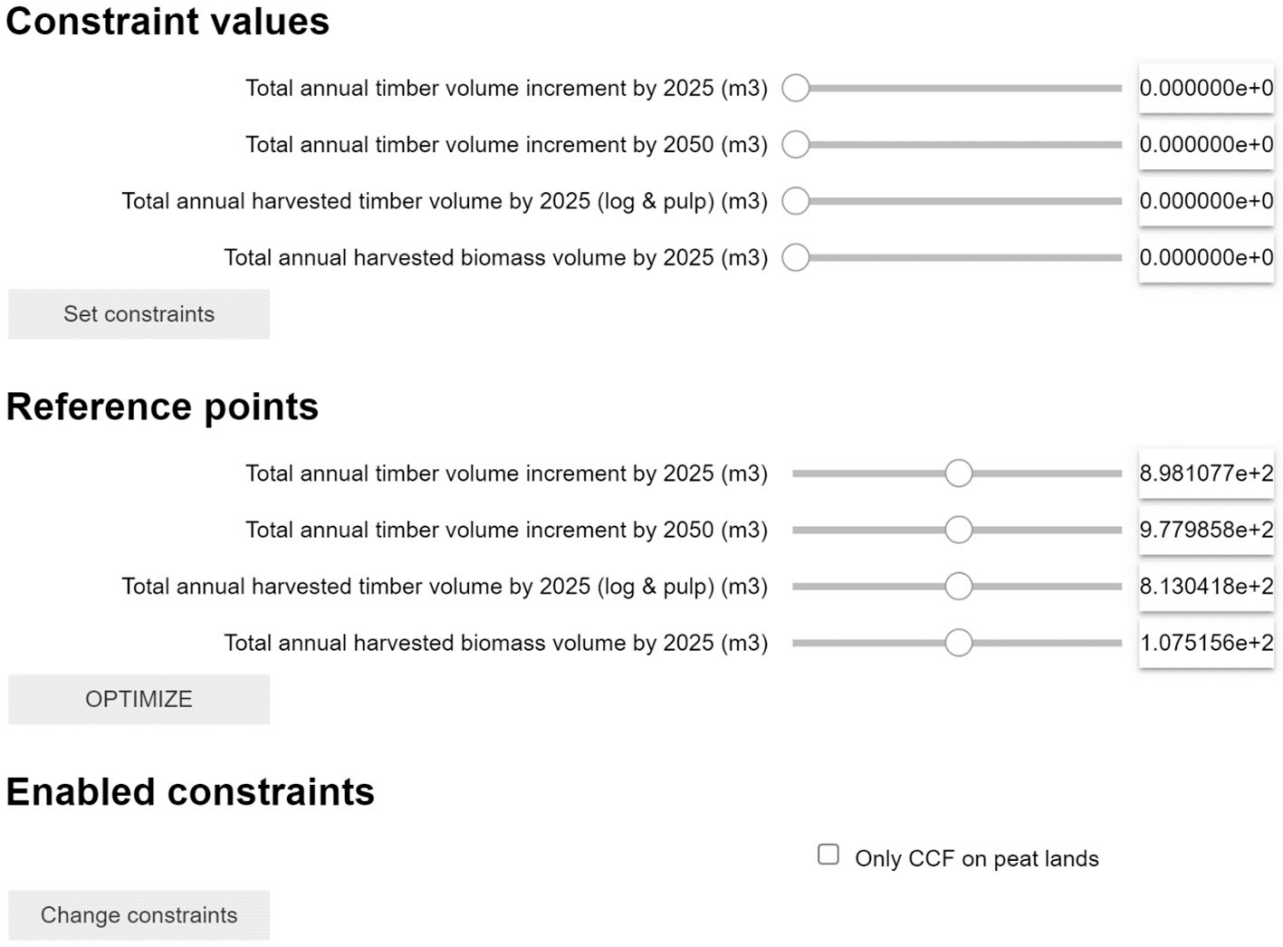
Example of the GUI for an optimization problem aiming to optimize forest management to reach four objectives. Each objective can be addressed as Constraint or Reference Point (or both) depending on the user's needs. Additionally, specific advanced constraints can be activated with the checkbox “Enabled constraint” (see 2.2.3).

The graphical user interface consists of three sections ( Figure 2). In the first section, named “Constraint values”, users can provide epsilon constraint values (requiring the solution to have values higher than the target). In the second section, named “Reference point”, users can provide aspiration levels for each objective (desired value for each objective). The aspiration levels jointly constitute a reference point from which a measure of preferability can be made. In the third section, “Enabled constraints”, users can include specified constraints to the decision problem (cf. Figure 3). In the first and second sections (“Constraint values” and “Reference point”), values can be set either by slider bars (see ”formulating objective functions”) or by typing the desired reference directly in the box ( Listing 2). In the third section, constraints can be enabled with a check box (see ”formulating constraints”). The ranges of the slider bars on the first and second sections are set to the anti-ideal and ideal values of each indicator, to allow the decision maker to set preferences that are feasible for the specific problem. To engage the additional defined constraints (Enabled constraints) requires a two-step process, first to click the check box and then to click the “Change constraints” box. Once the optimization is completed, options are available to export and visualize the results.

An example how the GUI can be loaded for the below described use case is described in the step-by-step user guideline for Code Ocean in the supplementary material (also linked on the Readme.md instructions).

**Figure 4.**
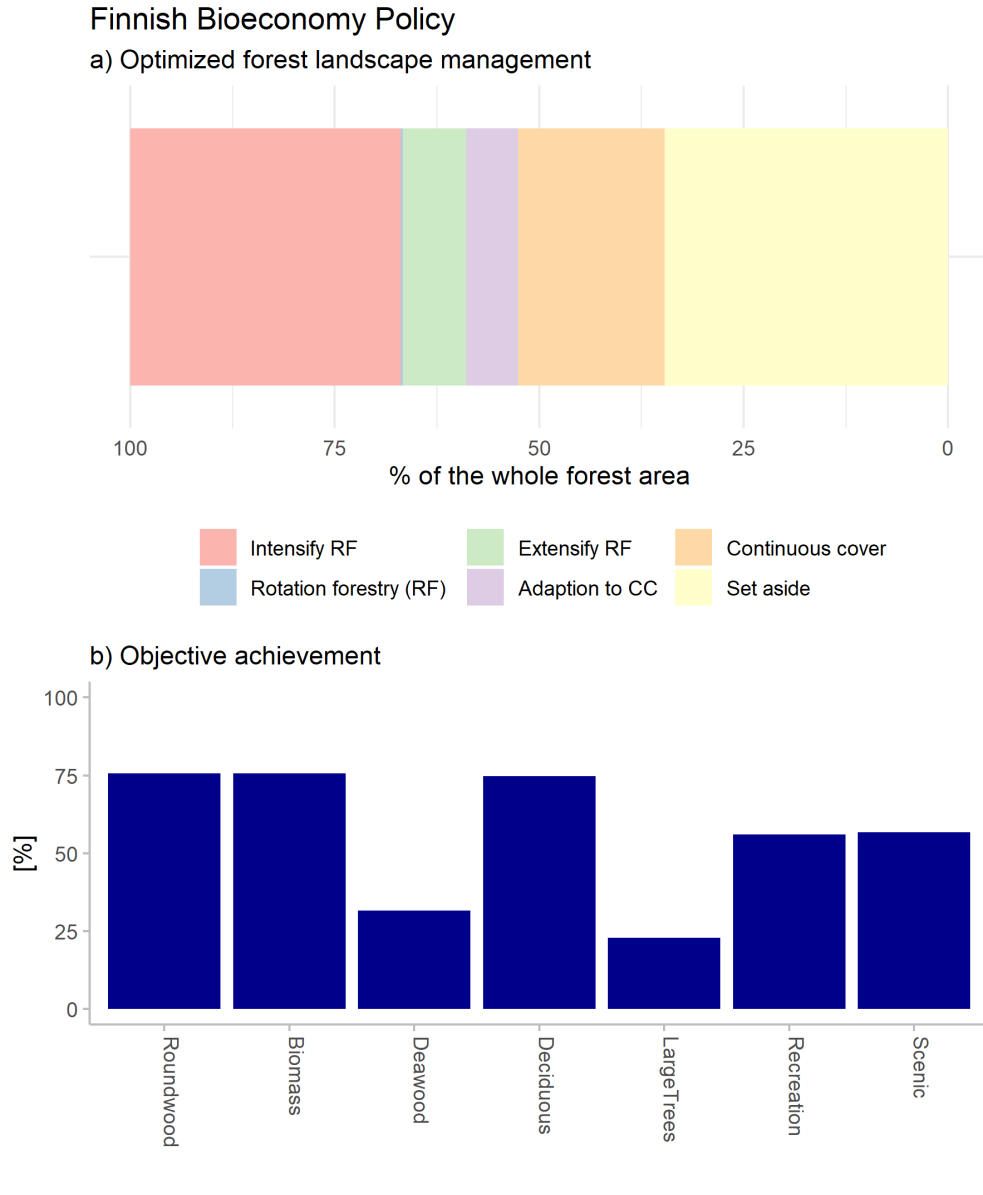
**a**) Optimal Forest management program that matches the societal demands for forest ecosystem service objectives of the Finnish Bioeconomy Policy under a single climate change scenario; **b**) achievement of the objectives, normalized among the ideal and anti-ideal value (maximum and minimum possible solution) (outcomes of alternative climate and policy scenarios are presented in Blattert *et al.*, 2022).

Data exportation means constructing text files for both the aggregate level and the stand level. The output is primarily a very large table indicating the management option selected for each stand, with a column to indicate how much of the stand is managed according to each specific management. This table includes all indicators produced by the forest simulator under the optimal management for each entity. This allows to produce a wide range further analysis and visualizations (comparable to Figure 4). To aid visualization, we currently have coded a selection of plots for optimal management portfolios and objective achievement plots (comparable Figure 4) as well as simple temporal line graphs, which can be explored on Code Ocean (see Supplementary information for a guideline of how to run the capsule in Code Ocean).

### Use case

The optimization tool was recently used to develop forest landscape management scenarios that match the multiple societal demands for forest ecosystem services stated in sectoral policies in Finland ( Blattert *et al.*, 2022), Norway ( Vergarechea *et al.*, 2023) and Germany ( Toraño Caicoya *et al.*, 2023). The modular character of the tool allowed to address the diverse objectives of national policies in a flexible way. For illustration, we describe one optimization problem that reflects the societal demands of the Finnish Bioeconomy Policy ( FMME *et al.*, 2014).

The Finnish Bioeconomy Policy anticipates a need for increased roundwood and biomass extraction to offset fossil fuels as a means to mitigate CO _2_ emissions that leads to a warming climate ( FMME *et al.*, 2014). The overall aim of the policy is to simultaneously mobilize forest resources for bioeconomy purposes while safeguarding biodiversity. The forest optimization problem strives to maximize an even-flow of harvested roundwood and biomass under the constraint that biodiversity indicators should not decline ( Table 2). Even flow was addressed by maximizing the minimum indicator value over all simulated years. Further, the objectives for the recreational value of forests were maximized as the policy also emphasized the importance of forest recreational value for society. Input data for the optimization was a Finland-wide systematic sample of forest stands ( FFC, 2021) that had been simulated with alternative management regimes representing even-aged rotation forest with final clearcut (data are available here) intensified and extensified versions of rotation forestry, regimes that foster adaptation against climate change, continuous cover forestry regimes and under set aside (without any intervention). The simulations were done using the SIMO forest simulation software and projected 100 years into the future under alternative climate scenarios (here presented for illustration purposes only for once climate scenario).

**Table 2.** Objectives and constraints that were defined and solved with the MultiOptForest software aiming to elaborate a forest landscape management that would match the societal demands of the Finnish Bioeconomy Policy (cf. Blattert *et al.*, 2022)).

	Indicator	Objective **(Obj)** / Constraint **(Con)**
**Wood production**	Harvested roundwood (m ^3^ ha ^-1^)	Maximise even-flow **(Obj)**
**Bioenergy**	Harvested biomass (m ^3^ ha ^-1^)	Maximise even-flow **(Obj)**
**Biodiversity** **Conservation**	Deadwood (m ^3^ ha ^-1^) Deciduous tree volume (%) Large trees (DBH > 40cm) (n ha ^-1^)	No decline allowed **(Con)** No decline allowed **(Con)** No decline allowed **(Con)**
**Recreation**	Recreation index (0-1) Scenic index (0-1)	Maximise **(Obj)** Maximise **(Obj)**

According to the results of the optimization, the policy objectives for the Bioeconomy Policy would require that approximately 2/3 of the forests would be managed by practices that include continuous cover forestry regimes and protected areas ( Figure 3). The remaining 1/3 of the forest should instead be intensively managed for wood production.

## Discussion

The MultiOptForest software simplifies the interactive assessment of complex forest planning challenges. Moreover, the software eases the building of multi-objective optimization problems and integration of new objectives for forest planning. This includes a rich and intuitive interface for formulating objectives and provides a common framework to explore the impact of management and targets for the forest using various sources of input data. The software user interface allows for interactive exploration of the set of Pareto optimal solutions using multi-objective optimization and constraint handling.

The MultiOptForest software was designed in a research project that aimed to critically compare the consistency between forest-related policy documents guiding the national/regional administration and management of forest ecosystem services and biodiversity ( Antón-Fernández *et al.*, 2022). At a national level in Finland ( Blattert *et al.*, 2022) and Norway ( Vergarechea *et al.*, 2023), at a regional level in Germany ( Toraño Caicoya *et al.*, 2023), and international level ( Blattert *et al.*, 2023) this software has been used to unveil synergies and conflicts between these government policies guiding forest use. With the help of this software, researchers were able to explore how forest management could be applied to balance conflicting objectives and best meet the stated policy goals in each country. The flexibility and functionalities of the software allowed to design a wide variety of objectives that match the diverse interpretations and contextual considerations of sustainable forest management state in these national policy documents.

This software has great potential to be used in a variety of forest planning and forest policy development research, as the tool constructs objective functions in a systematic fashion and allows for the comparison of alternative multi-objective optimization scenarios. While the optimization framework can use simulation data from multiple sources, this software integrates with nationally/regionally specific forest management software to enable nationally/regionally relevant scenarios. Although the current use of the software has focused on very large-scale problems, it can be applied at smaller spatial scales, such as forest holdings, or certain landscapes. The computational time and complexity however depend on the number of individual optimized entities (forest plots or stands). The software has not yet been used in commercial settings, however forest planners could integrate this as an iterative approach to improve forest planning.

Due to the simplicity of its user interface, the MultiOptForest has the potential to become a tool to help untrained users to understand the conflicts among forest ecosystem services and biodiversity. This could be used, for instance, in participatory workshops seeking consensus solutions where forest stakeholders from different sectors examine the consequences of their preferences. The nested construction from simpler to more complex multi-objective optimization problems also allow to use the tool for educational purposes. Nevertheless, in the current version still certain IT skills are required to use the optimization tool. The provided software capsule on Code Ocean together with the short user guide however aims to ease the first steps with the tool avoiding complex pre-installations.

## Conclusions

The key benefit of the MultiOptForest software is the flexibility to define the specific objectives of interest to the decision maker. The software utilizes theoretically sound multi-objective optimization techniques, packaged in a user-friendly software package with which decision-makers can interact. The easy-to-understand interface enables users with no training in coding to modify preferences, evaluate different optimized scenarios and gain an understanding of the trade-offs and synergies involved in their decisions.

## Data Availability

Input data for the use case presented (and example cases from Germany, Sweden and Norway) is available in the data folder in: https://doi.org/10.5281/zenodo.7885954. And is originally available from https://doi.org/10.5281/zenodo.6631110 ( Blattert *et al.*, 2022a) under a CC-BY 4.0 license.

## Software availability

**Software available from:**

**Source code:** https://codeocean.com/capsule/2689439

**Archived source code at the time of publication:** https://doi.org/10.5281/zenodo.7885953 ( Eyvindson *et al.*, 2023)

**License:** MIT
