# Supplementary material for: MultiOptForest: An interactive multi-objective optimization tool for forest planning and scenario analysis
Source: Open Res Eur. 2024 Dec 16;3:103. Originally published 2023 Jun 21. [Version 2] doi: 10.12688/openreseurope.15812.2 (PMC11809471; doi:10.12688/openreseurope.15812.2)
Supplement: Peer review history

# Reviewer report: Reviewer response for version 2

**DOI:** 10.21956/openreseurope.20653.r48766

**Published:** 2025-01-10

**Authors:** Logan Robert Bingham

**Affiliations:** 1 Technical University of Munich, Hans-Carl-von-Carlowitz-Platz, Freising, Germany

The manuscript “ *MultiOptForest: An interactive multi-objective optimization tool for forest planning and scenario analysis" *introduces an open-source decision support tool that uses multi-objective optimization to support the incorporation of ecosystem services into forest management planning. The manuscript has already gone through one round of review. The points raised by the other referees struck me as overall useful and relevant; I'll leave it to them to determine whether the revision has resolved their concerns. Instead, I'd like to offer another perspective: I found this manuscript useful for the work I'm doing right now, and I expect the same will be true for others in my field, so I think it should be indexed.

 While there's always room to refine the description and add more detail, the main contribution is to introduce software architecture and usage to a primarily scientific readership—not necessarily to undertake an exhaustive theoretical analysis or present a detailed user manual in the main text (the online supplementary materials contain additional detail and example problems). I think it succeeds. Others might reasonably disagree, but given the state of the software and the manuscript, I think it's appropriate for that discussion to move beyond the peer review process and into the broader scientific community. Relatively approachable open-source Pareto frontier tools for forest management are not exactly in great abundance; I think this one should be disseminated so that a wider audience can experiment with different applications, especially since work to further develop the software and expand the GUI is ongoing.

 Below, I make a few minor comments the authors can incorporate or not at their own discretion.

**Minor comments**

 1. Manage reader expectations

 Several of the reviewers' previous comments involved the usability of the software and how much expertise is required. In the revision, the authors helpfully distinguish between which actions are available to non-expert users and which require some coding knowledge. That's fair, but I have to confess that I also felt a bit confused by this point on my first read. Maybe the first paragraph of the introduction could do a better job of managing reader expectations? The reader gets the impression that the software meets all of the points (a-e) simultaneously, which isn't quite the case. For example, I don't think it's the case that users with limited computer skills (point a) can integrate different growth simulators (point c) or different objectives (point e); these require more advanced scripting and/or forest management knowledge. The authors might consider rephrasing or adding some qualification here to better calibrate  reader expectations. I think this might help clear up some of the previous comments.

 2. Clarifying the role of expertise

 In a similar vein, later in the introduction the authors note that advanced users can connect forest simulator data, while "non-expert" users like policymakers and NGOs can explore scenarios by playing around with the GUI. Having clicked through the example case studies in the Jupyter notebook, I'm not quite sure that "non-expert" is the best designation. While it's true the GUI allows users who can't code to adjust preferences and run the model, it seems to me that some expertise is needed to understand the sliders and the trade-offs in the first place. For instance, the ranges of some sliders are fairly large, and quite small for others, units vary, and interpreting the trade-offs does seem to require some subject matter expertise (even if the user doesn't need to be familiar with Python to manipulate the actual sliders). Maybe the expert vs non-expert distinction would be better phrased as "scripting users vs GUI users" or "technical users vs basic users"? Alternatively, the authors might set aside the issue of user abilities entirely and just focusing on the software itself: i.e., remove the stuff about experts and non-experts and just keep descriptions like  "In the current version of the software, the GUI interface allows users to do ABC, but users who are familiar with Python syntax can additionally perform actions XYZ."

 3. A few minor typos/language issues

 p4 para 1: "... optimal management scenarios and conflicts or trade-off between" should be "trade-offs"

 p4 para. 3: RAM usually capitalized

 p4 middle right: --> "Components six and seven relate to quantifying user preferences" or something along those lines?

 p4 right, Formulating additional constraints: "The majority of the components should be formulated as optimization formulations and epsilon constraints" Do you mean "formulated as objective functions and epsilon constraints"?

 p. 8 "Use case": Maybe "The modular character of the tool allows diverse policy objectives to be addressed in a flexible way"?

 Fig. 4a caption: "ideal and anti-ideal" Wasn't the term used previously ideal and nadir?\

Is the rationale for developing the new software tool clearly explained?

Yes

Is the description of the software tool technically sound?

Yes

Are sufficient details of the code, methods and analysis (if applicable) provided to allow replication of the software development and its use by others?

Partly

Is sufficient information provided to allow interpretation of the expected output datasets and any results generated using the tool?

Yes

Reviewer Expertise:

Ecosystem services, forest management, trade-off analysis, decision support systems

I confirm that I have read this submission and believe that I have an appropriate level of expertise to confirm that it is of an acceptable scientific standard.

# Reviewer report: Reviewer response for version 2

**DOI:** 10.21956/openreseurope.20653.r48764

**Published:** 2024-12-26

**Authors:** Yuxiang Dong

**Affiliations:** 1 Tongji University, Shanghai, Shanghai, China

1. summary of the article

 The software proposed demonstrates academic value and overall performs well in its intended purpose. For me the primary contribution of this paper is its attempt to develop an interactive model that engages a broader audience, including non-experts, to explore varying preferences for optimal solutions. Also, the compatibility with multiple simulators and potential optimization solvers represents another innovative aspect. 

 While the model shows promise, further simplification and enhanced flexibility may be necessary to facilitate its direct application in a broader range of contexts.

 I have reviewed not only the current version of the paper but also the reviewer comments from the previous round revision. The paper shows notable  improvements, but I'd like to offer some further suggestions for authors' consideration.

 2. Detailed suggestions for the question: Are sufficient details of the code, methods and analysis (if applicable) provided to allow replication of the software development and its use by others?

 a. I found several reviewers from the previous round raised concerns about the input requirements, to which the authors responded by adding descriptions. However, I still found them insufficient to fully address the concern. I  understand that clarifying the input requirements is challenging at this stage as many variables appear to be hardcoded within the existing codebase. For example, upon reviewing the source code, I found several lines used for defining regime names as follows:

 ```

 # snippets from Optimization_FIN.py

 self.regimeClassNames = {"regimeClass0name":"CCF","regimeClass1name":"SA","regimeClass2name":"Broadleave"}

 self.regimeClassregimes = {"regimeClass0regimes":["CCF_3","CCF_4","BAUwGTR"],"regimeClass1regimes":["SA"],"regimeClass2regimes":["BAUwT_B", "BAUwT_5_B", "BAUwT_15_B", "BAUwT_30_B", "BAUwT_GTR_B"]}

 # snippets from Optimization_GER.py

 self.regimeClassNames = {"regimeClass0name":"CCF","regimeClass1name":"SA"}

 self.regimeClassregimes = {"regimeClass0regimes":["CCF_P3 ","CCF_P3_p1","CCF_P3_p2"],"regimeClass1regimes":["NOT"]}

 ```

 Similarly, both constraints and objectives are currently hardcoded. Hardcoding may not be a practical or flexible way for a universal tool intended for non-experts. A potential way could involve modifying the code to identify variables automatically from the input files. For example, users could be instructed to reorganize their input table into columns labeled `id(of stands)`, `year`, `regime`, and other targets. Regimes could be formatted to follow specific patterns such as those beginning with `CCF` or `BAU`. Using such patterns, column names can be easily identified as regime or target names in Python, enabling the GUI to automatically update based on the input. Implementing this feature could enhance the model's usability, as many non-experts are likely to prefer uploading their results directly and interacting with the visual interface without additional coding tasks.

 I understand this tool is still under development, and may not be updated immediately, so I suggest the authors either discuss universal input processing as a future task or provide justification for retaining the tool in its current form. 

 b. Traditionally, a posteriori methods require obtaining the entire Pareto frontier, whereas interactive methods seem to adopt a logic that lies between a priori and a posteriori approaches. Some readers, myself included, may initially wonder why the authors have not provided a tool for plotting the entire Pareto frontier. I suggest that the authors briefly introduce the theory behind the interactive multi-objective optimization method and explain how it differs from traditional a priori and a posteriori methods in the introduction. Additionally, it may be helpful to provide a clear explanation in the main text on how Pareto solutions and trade-offs can be explored using the interactive method, as this objective was mentioned in the introduction. 

 3. Minor issues

 a. The section named "formulating objective functions and epsilon constraints", is sometimes referred as section "formulating objective functions". Same for section named "formulating additional constraints". Please make them consistent. 

 b. The abbreviation 'DBH' in table 2 was not mentioned and explained earlier in the text.

 c. The conclusion describes the tool as "packaged in a user-friendly software package," which may be an overstatement. A Jupyter Notebook-based GUI might not fully qualify as a packaged software. I suggest revising this statement for greater accuracy and rigor.

Is the rationale for developing the new software tool clearly explained?

Yes

Is the description of the software tool technically sound?

Yes

Are sufficient details of the code, methods and analysis (if applicable) provided to allow replication of the software development and its use by others?

Partly

Is sufficient information provided to allow interpretation of the expected output datasets and any results generated using the tool?

Yes

Reviewer Expertise:

Multi-objective optimization, ecosystem service, green infrastructure

I confirm that I have read this submission and believe that I have an appropriate level of expertise to confirm that it is of an acceptable scientific standard, however I have significant reservations, as outlined above.

# Reviewer report: Reviewer response for version 1

**DOI:** 10.21956/openreseurope.17082.r35235

**Published:** 2023-11-02

**Authors:** Olalla Díaz-Yáñez, Sina Heubel

**Affiliations:** 1 Forest Resources Management - Department of Environmental Systems Science, Eidgenossische Technische Hochschule Zurich, Zürich, Zurich, Switzerland

The software tool article “MultiOptForest: An interactive multi-objective optimization tool for forest planning and scenario analysis” describes the open-source software MultiOptForest. This software is designed to simplify the process of building and solving multi-objective optimization problems that consider various ecosystem services. The tool has been tested in several case studies in Fennoscandia and Germany.

 Even though there are several software applications available that can provide Pareto optimal solutions and illustrate trade-offs among multiple objectives, most of these software tools are either not open-source or lack clear instructions regarding their usability. Therefore, we strongly believe that there is a clear justification for creating a new software tool that is open-source and can be easily accessed by all.

 We think that the software tool article and its related materials hold academic value, although we have a few minor suggestions that we would like the authors to consider. Specifically, we have some comments and suggestions on how the software's description could be improved. Additionally, we believe that some aspects of the article need further elaboration, as they are not clear enough from the current text. Please refer to our detailed comments below for further clarification.

 The article provides detailed information about the code and methods used in the software, which can be replicated by others. It also includes four examples that were used in other publications as supplementary materials. Although the authors have provided a text readme, we believe that a visual step-by-step guide, such as a vignette, could be added to facilitate the understanding of these examples. 

 We also think that a more detailed description of the software code in the article text, such as adding a pseudocode, would benefit the readers. This will help them understand the different parts of the routine, the flow between the different steps, and explain some aspects of the text more clearly.

 The information provided allows interpretation of the approach and results via one example in the article and more in the appendix materials. However, we would appreciate it if the example in the text could be explained more thoroughly. Please refer to our detailed comments below for further clarification.

**Detailed comments:**

**Rationale: **The authors of the paper mentioned the importance of having a general optimization tool that is easy to use, even for users who have limited computer skills. However, they also acknowledged that to use this tool, advanced user skills are required, such as for determining appropriate optimization objectives. Although a user interface can help visualize trade-offs under different preferences, there are still previous steps in using this tool that are not intuitive for all users. We recommend that the discussion section address this point as well.

**Description of the software tool - The input requirements: **In the description of the software tool, it was initially challenging to understand the input requirements needed by the optimization tool when they were first introduced in the paragraph: “The input requirements of this software are projections of multiple alternative management…”. To make it clearer, a concrete example could be added early on, perhaps in the same paragraph, with a reference to the section where the formulation of objective functions is described. For instance, "The user can define the individual optimization objectives based on the simulator outputs, e.g., for biodiversity, the user may choose the relative amount of deadwood at a specific year."

**Description of the software tool - The eight standardized attributes: **We want to make sure we understand correctly: the user is required to calculate standardized attributes based on the outputs of their simulators. The reason for the minimum standard set of information from the forest simulators is to enable the estimation of the eight selected attributes, correct? We believe that further clarifying these two aspects would benefit the reader.

**Description of the software tool - The preference information: **Is the preference information provided as a weight to the objective function? Is the user defining these weights? Please clarify this also in the text.

 Example provided: In the article, an example is presented, and the results are displayed in Figure 3, which illustrates the Finnish case study. Although this example is from a published study, it would be helpful to provide more information about the context. A more detailed caption could be added, specifying whether the optimization was spatially explicit or conducted under different climate scenarios. It is unclear how the user of the tool can analyze the Pareto optimal solution space from this example. It would be beneficial to see this aspect of the tool's functionality demonstrated in the example, as it is one of the tool's strengths.

**Minor comments:**

**Text:**

- “however for the example cases found in the source code (Eyvindson et al., 2023, or from Code Ocean) were able to be run with a virtual machine running Ubuntu 18.04, with 3 Gb of ram.” → Grammar → "However, the example cases found in the source code (Eyvindson et al. (2023), or from Code Ocean) were able to be run on a virtual machine running Ubuntu 18.04 with 3 GB of RAM."
- “Our software has been tested to import data from four forest simulators” → add which forest simulators
- “Data can be provided from a variety of different forest simulators, but a minimum standard set of information from all forest simulators is required, indexed data (stand ID, year, management regime) and indicators representing the state of the forest and ecosystem services (country or model specific)” → The period at the end of the sentence is missing

**Figures, Listings, and Tables:**

- **Figure 1: **
  - What does this part of the caption refer to and what does it mean in the caption of this figure: “T2.1 Accounting for risks and uncertainties in forest-based businesses, sectoral projections, and policy design.”
  - In box “interface”: reference to Figure 4 but Fig 4 not available in article → should be Figure 2 (?)
  - In box “Optimization”: reference to Figure 2 → example for GUI rather than optimization code
- **Table 1:** Improve this caption: “Table 1. Approaches in how the temporal aspects can be handled in the MultiForestOpt software, for each coded interpretation of how temporal aspects are currently implemented.”

**Other:** The data and descriptions thereof for the applied Finnish use case are not available in English, which makes it more complicated to reproduce and understand.

Is the rationale for developing the new software tool clearly explained?

Yes

Is the description of the software tool technically sound?

Partly

Are sufficient details of the code, methods and analysis (if applicable) provided to allow replication of the software development and its use by others?

Yes

Is sufficient information provided to allow interpretation of the expected output datasets and any results generated using the tool?

Partly

Reviewer Expertise:

Forest dynamics, forest optimization, forest management and planning

We confirm that we have read this submission and believe that we have an appropriate level of expertise to confirm that it is of an acceptable scientific standard, however we have significant reservations, as outlined above.

# Response

**Published:** 2024-12-02

**Authors:** Kyle Eyvindson

**Affiliations:** MINA, Norges miljø- og biovitenskapelige universitet, Ås, Norway

**Authors response:**

 We appreciate the constructive review. To respond to the suggestions and comments, we respond to the reviewer directly. **Reviewer comments:**

 The software tool article “MultiOptForest: An interactive multi-objective optimization tool for forest planning and scenario analysis” describes the open-source software MultiOptForest. This software is designed to simplify the process of building and solving multi-objective optimization problems that consider various ecosystem services. The tool has been tested in several case studies in Fennoscandia and Germany. Even though there are several software applications available that can provide Pareto optimal solutions and illustrate trade-offs among multiple objectives, most of these software tools are either not open-source or lack clear instructions regarding their usability. Therefore, we strongly believe that there is a clear justification for creating a new software tool that is open-source and can be easily accessed by all. We think that the software tool article and its related materials hold academic value, although we have a few minor suggestions that we would like the authors to consider. Specifically, we have some comments and suggestions on how the software's description could be improved. Additionally, we believe that some aspects of the article need further elaboration, as they are not clear enough from the current text. Please refer to our detailed comments below for further clarification.

**Authors response:**

 Thank you for the constructive comments, we generally agree with the vast majority of the suggestions, and have integrated them when possible into the article. **Reviewer comments:**

 The article provides detailed information about the code and methods used in the software, which can be replicated by others. It also includes four examples that were used in other publications as supplementary materials. Although the authors have provided a text readme, we believe that a visual step-by-step guide, such as a vignette, could be added to facilitate the understanding of these examples. **Authors response:**

 When responding to Reviewer 2, we have updated the software architecture section of the manuscript to better describe the flow and steps of the optimization process. We hope that this adjustment also satisfies this reviewer. Additionally, we have generated a step-by-step user guide illustrating with screen shots how the tool can be virtually launched via Code Ocean, providing as such an illustrative example how user can get familiar with the tool: How to start the tool on Code Ocean, how to Launch Jupiter notebook, selecting one of the country example, and run the Jupyter Notebook, which loads the GUI where constraints/objective can be set. (see also our response to Reviewer #1). The step-by-step user guide can be found in the supplementary material and is linked to the Readme.md on Code Ocean.

**Reviewer comments:**

 We also think that a more detailed description of the software code in the article text, such as adding a pseudocode, would benefit the readers. This will help them understand the different parts of the routine, the flow between the different steps, and explain some aspects of the text more clearly. **Authors response:**

 We have revised the software architechture section including a new section on “Individual components” followed by the exisitng part under the new defined section “Levels of software organization” (see also our response to R#2). A more detailed description of the software code we have refrained, as we believe this would make the article to complex. All code is transparent and open source via code ocean allowing even an “online exploration of the funktionalities without any software installtions, as well as on the git Repository.  

 For the flow between the different steps we created a detailed step-by-step user guide for Code Ocean, which can be found in the Supplementary material (see our comment above). 

**Reviewer comments:**

 The information provided allows interpretation of the approach and results via one example in the article and more in the appendix materials. However, we would appreciate it if the example in the text could be explained more thoroughly. Please refer to our detailed comments below for further clarification.

**Authors response:**

 To improve the description of the software code, we have thoroughly updated Figure 1 – which now presents the architecture of the software. While this isn’t exactly Pseudocode – it does more clearly highlight what is happening with the different components of the code.  

**Reviewer comments:**

 Detailed comments:

 Rationale: The authors of the paper mentioned the importance of having a general optimization tool that is easy to use, even for users who have limited computer skills. However, they also acknowledged that to use this tool, advanced user skills are required, such as for determining appropriate optimization objectives. Although a user interface can help visualize trade-offs under different preferences, there are still previous steps in using this tool that are not intuitive for all users. We recommend that the discussion section address this point as well.

**Authors response:**

 We agree that there is a combination of advanced user skills required – which is required to adjust the code to enable data imputation and modifications to the selected objective functions. However, from a user experience, we aim to ease the optimization process as much as possible. We reflect on this in the discussion section. See the last sentence of the last paragraph in the discussion.

**Reviewer comments:**

 Description of the software tool - The input requirements: In the description of the software tool, it was initially challenging to understand the input requirements needed by the optimization tool when they were first introduced in the paragraph: “The input requirements of this software are projections of multiple alternative management…”. To make it clearer, a concrete example could be added early on, perhaps in the same paragraph, with a reference to the section where the formulation of objective functions is described. For instance, "The user can define the individual optimization objectives based on the simulator outputs, e.g., for biodiversity, the user may choose the relative amount of deadwood at a specific year."

**Authors response:**

 We agree with this, and we hope that the restructuring of figure 1 helps with this. Additionally, we have revised the input requirements in the introduction section to: “The user can define the individual optimization objectives based on the simulator outputs, for instance as an indicator for biodiversity the user may strive to maximize deadwood at a specific year. Additionally, the objective functions should be based on the interest and ability of the forest owner to provide preference information.” Additionally, we aimed to highlight the advantages of the open source example provided on Code Ocean, which aims to ease first application steps (see also comments above)

**Reviewer comments:**

 Description of the software tool - The eight standardized attributes: We want to make sure we understand correctly: the user is required to calculate standardized attributes based on the outputs of their simulators. The reason for the minimum standard set of information from the forest simulators is to enable the estimation of the eight selected attributes, correct? We believe that further clarifying these two aspects would benefit the reader.

**Authors response:**

 The attributes are more related to the ‘advanced user’, although we are working on constructing a GUI component where a normal user can construct the objectives. We currently limit the construction of objectives to more advanced users to ensure that the logic of the objectives are reasonable. The set of information is mainly to define how the objective is constructed. The user doesn’t need to standardize anything – this is done through the payoff tables that were constructed in the code. We removed the word “standardized” to avoid misunderstandings. Additionally, we added the sentence, that “Based on these attributes the more advanced user is able to build individual objective functions depending on the optimization targets.”

**Reviewer comments:**

 Description of the software tool - The preference information: Is the preference information provided as a weight to the objective function? Is the user defining these weights? Please clarify this also in the text.

**Authors response:**

 The preference information is provided as reference values and shouldn’t be interpreted as weights. We have opted to use the achievement scalarizing function, that uses reference points as a direction. The reference points should be interpreted as “the value of the specific objective where the user is satisfied”. We have opted to not use weights, as it can be difficult to understand what the weights mean in an optimization sense.

 When updating Figure 1, we have discussed what the preference information is “provided as target values (objectives) or minimum threshold values (constraints)”

**Reviewer comments:**

 Example provided: In the article, an example is presented, and the results are displayed in Figure 3, which illustrates the Finnish case study. Although this example is from a published study, it would be helpful to provide more information about the context. A more detailed caption could be added, specifying whether the optimization was spatially explicit or conducted under different climate scenarios. It is unclear how the user of the tool can analyze the Pareto optimal solution space from this example. It would be beneficial to see this aspect of the tool's functionality demonstrated in the example, as it is one of the tool's strengths.

**Authors response:**

 We have added some additional information into the caption of figure 4 (now after revisions), and in the text. For this case, we are only exploring a single future scenario (so one climate change scenario), without uncertainty.

 Figure 4 does show the results of one case study and one climate scenario, and doesn’t assess trade-offs/Pareto frontiers between objectives. This wasn’t the focus of the optimization approach, nor was this the focus of the software. However, this software could be used to assess trade-offs, if desired. This would however require storing a variety of solutions to enable comparisons. To visualize trade-offs between preference sets, you would need to extract solutions for each preference set, and compare. However, from our perspective, multi-objective optimization was used to construct preference specific solutions, and not specifically to quantify and evaluate trade-offs. (please see also our response to R#2 comment 4)

**Reviewer comments:**

 Minor comments: 

 Text:

 •    “however for the example cases found in the source code (Eyvindson et al., 2023, or from Code Ocean) were able to be run with a virtual machine running Ubuntu 18.04, with 3 Gb of ram.” → Grammar → "However, the example cases found in the source code (Eyvindson et al. (2023), or from Code Ocean) were able to be run on a virtual machine running Ubuntu 18.04 with 3 GB of RAM."

 •    “Our software has been tested to import data from four forest simulators” → add which forest simulators

 •    “Data can be provided from a variety of different forest simulators, but a minimum standard set of information from all forest simulators is required, indexed data (stand ID, year, management regime) and indicators representing the state of the forest and ecosystem services (country or model specific)” → The period at the end of the sentence is missing

 Figures, Listings, and Tables:

 •    Figure 1: 

 - What does this part of the caption refer to and what does it mean in the caption of this figure: “T2.1 Accounting for risks and uncertainties in forest-based businesses, sectoral projections, and policy design.”

 •    - In box “interface”: reference to Figure 4 but Fig 4 not available in article → should be Figure 2 (?)

 •    - In box “Optimization”: reference to Figure 2 → example for GUI rather than optimization code

 •    Table 1: Improve this caption: “Table 1. Approaches in how the temporal aspects can be handled in the MultiOptForest software, for each coded interpretation of how temporal aspects are currently implemented.” **Authors response:** We agree, and have edited these minor adjustments as required. We have included a description of the forest simulators used in the development of this software. We have adjusted the figures to refer to the correct aspects. And have improved the caption of table 1 to: ”Table 1. Advanced users can handle temporal aspects in MultiOptForest when constructing individual objective functions. Each coded interpretation specifies how to implement these temporal aspects when solving the optimization problem” **Reviewer comments:**

 Other: The data and descriptions thereof for the applied Finnish use case are not available in English, which makes it more complicated to reproduce and understand. **Authors response:**

 Example and data on code ocean are in English.

# Reviewer report: Reviewer response for version 1

**DOI:** 10.21956/openreseurope.17082.r35230

**Published:** 2023-11-02

**Authors:** Marc McDill, Silvana Nobre

**Affiliations:** 1 Penn State University, State College, PA, USA; 2 ETS Ingeniería de Montes, Forestal y del Medio Natural, University of Madrid, Madrid, Spain

The article describes a software system that can be used to formulate and solve multi-objective forest management problems. The software is undoubtedly an excellent product for the developers to use for their research, but the article does not provide sufficient information for new users who might be interested in using the software. Furthermore, the article would not be particularly helpful to developers interested in creating similar applications.

**Major comments: System "Architecture"**

 The article identifies two types of users of MultiOptForest: advanced users and non-expert users. It seems that most applications would involve both types of users. It would be helpful to have some kind of flow chart that shows the process of building a MultiOptForest problem instance and what the roles are of these different users. Such a flow chart should start from the beginning (creating input files) and guide the reader through to the point where outputs are produced. It would indicate the inputs and outputs from the system, where and how users provide input, where and how output is provided, and what happens in between. This is what I think "software architecture" is about. Figure 1, unfortunately, does not help with understanding these things.

 Regarding inputs, it would be helpful to describe more specifically what inputs are required and how they are input into the system. The article is very vague on this point, indicating only that one would need and "advanced user" to understand these things. The article should indicate what inputs are provided through files that are read by the system and how these files must be structured. Also, which inputs are provided through the GUI, and at what point in the process does this occur? There is a paragraph on importing data, but this paragraph does not indicate specifically what data are needed by the system, only what kind of systems have been used to generate input data. If I wanted to use a different system (or even one of those listed) what would I need to do to create input files for MultiOptForest? 

 In the middle of the system (somewhere) an (or more likely many...) optimization problem is formulated and solved. The article discusses how different solvers can be used, but, again, what would I have to do if I wanted to use Gurobi - for instance - within MultiOptForest?

 On the output side, the article offers three vague sentences about the program's output. I would like to know more about what is included in the output. Does it look something like Figure 3? How might the output look for a different type of forest, perhaps with different management options and different objectives? What if I want more detail about a given Pareto optimal solution? How can I visualize trade-offs among different preference sets? Figure 3 shows results for one set of preferences from the case study, but it does not illustrate how the system was used to assess trade-offs between different objectives, which, to me, is a key point of multiobjective optimization.

**Major comments: Constraints vs Objectives**

 The article is not clear on the distinction between constraints and objectives. For example, one item listed as an objective in the section "Formulating objective functions" sounds more like a constraint than an objectives. In particular, "increase the quantity of deadwood in the forest by a specific year, and to ensure that quantity is kept for the rest of the planning horizon" sounds like a constraint to me. Furthermore, the software offers a dozen ways to create an objective but only (apparently) two ways to create a constraint. In the case study (see Table 2), objectives and constraints are mixed together with no indication which items are objectives and which are constraints.

**Minor comments:**

 The article states that: "Forest planning literature often focuses on the production of timber resources, obtaining a relative even-flow of timber over time while minimizing costs of logistics and maximizing timber production." This is not accurate. There is a large literature of forest planning models that address multiple objectives.

 The article references two similar packages, desdeo-mcdm and optimLanduse, but it does not elaborate on what the similarities and differences are between MultiOptForest and these other packages. For example, could one use either desdeo-mcdm and optimLanduse instead of MultiOptForest?

 I would like to acknowledge the assistance of Silvana Nobre in developing this review.

Is the rationale for developing the new software tool clearly explained?

Yes

Is the description of the software tool technically sound?

Partly

Are sufficient details of the code, methods and analysis (if applicable) provided to allow replication of the software development and its use by others?

No

Is sufficient information provided to allow interpretation of the expected output datasets and any results generated using the tool?

Partly

Reviewer Expertise:

Forest economics, forest management, forest management planning, optimization, forest ecology, remote sensing of forests

We confirm that we have read this submission and believe that we have an appropriate level of expertise to state that we do not consider it to be of an acceptable scientific standard, for reasons outlined above.

# Response

**Published:** 2024-12-02

**Authors:** Kyle Eyvindson

**Affiliations:** MINA, Norges miljø- og biovitenskapelige universitet, Ås, Norway

**Authors response:** We appreciate the constructive review. To respond to the suggestions and comments, we respond to the reviewer directly.

**Reviewer comments: **The article describes a software system that can be used to formulate and solve multi-objective forest management problems. The software is undoubtedly an excellent product for the developers to use for their research, but the article does not provide sufficient information for new users who might be interested in using the software. Furthermore, the article would not be particularly helpful to developers interested in creating similar applications. Major comments: System "Architecture"

 The article identifies two types of users of MultiOptForest: advanced users and non-expert users. It seems that most applications would involve both types of users. It would be helpful to have some kind of flow chart that shows the process of building a MultiOptForest problem instance and what the roles are of these different users. Such a flow chart should start from the beginning (creating input files) and guide the reader through to the point where outputs are produced. It would indicate the inputs and outputs from the system, where and how users provide input, where and how output is provided, and what happens in between. This is what I think "software architecture" is about. Figure 1, unfortunately, does not help with understanding these things.

**Authors response: **We thank the reviewer for this constructive feedback. When constructing the ‘architecture’, we were considering the levels of abstraction that were being made in the code – however, as the reviewer indicates is not very informative. To address this, we have revised figure 1, to reflect the architecture of the software in a more sensible fashion, highlighting the core components of the optimization tool. Those we grouped along: the required input, processing the data and output from the optimization framework, with the key tasks performed at each step. We have also revised software architecture section to reflect the changes including a new section on “Individual components” followed by the existing part under the new defined section “Levels of software organization”.

**Reviewer comments: **Regarding inputs, it would be helpful to describe more specifically what inputs are required and how they are input into the system. The article is very vague on this point, indicating only that one would need and "advanced user" to understand these things. The article should indicate what inputs are provided through files that are read by the system and how these files must be structured. Also, which inputs are provided through the GUI, and at what point in the process does this occur? There is a paragraph on importing data, but this paragraph does not indicate specifically what data are needed by the system, only what kind of systems have been used to generate input data. If I wanted to use a different system (or even one of those listed) what would I need to do to create input files for MultiOptForest? 

**Authors response: **We thank the reviewer for highlighting this issue. We wanted the input data to be as flexible as possible, so that input data could be from any forest simulator. Of course, the optimization problem requires a standardized data format, however this format should be possible to create using data processing techniques. Currently, the data is structured as a table – with example datasets provided in the Code Ocean test data. However, as long as a table can be structures with the indices of “stand”, “management style” and “year”, the optimization software can construct relevant (user defined) objectives and constraints.

 To address this issue, we have reorganized figure 1 to use a “input-process-output“ format. We anticipate that expert users will structure the input data, and frame the objectives and constraints, while normal users can interact with the GUI. Currently, only the user preferences information is provided through the GUI. We are considering options to enable user construction of objectives and constraints, however currently they are accessible to moderately advanced coders.

 If you wish to use a different forest simulator – the data currently needs to be structured as it is in the examples, or the code needs to be edited so that the data is modified to reflect that structure. The structure is a table, with indices for the ‘stand’, ‘management style’ and ‘year’ – all of the columns are then variables (e.g. ecosystem service and biodiversity indicators) that describe that specific stand, according to that management style at that specific time period.

 We added a paragraph to the “importing data” section saying: Examples for the importing data of the above four forest models are on Code Ocean and can be interactively loaded for the below use case following the step-by-step guide included in the supplementary material and linked on the Readme.md instructions (Getting started).

**Reviewer comments: **In the middle of the system (somewhere) an (or more likely many...) optimization problem is formulated and solved. The article discusses how different solvers can be used, but, again, what would I have to do if I wanted to use Gurobi - for instance - within MultiOptForest?

**Authors response: **There are a few optimizations that are conducted to enable the use of the achievement scalarizing function (to estimate the nadir and ideal values of each objective), however at the end, the interactive optimization problem is a single multi-objective optimization function using the achievement scalarizing function with epsilon constraints (with an option to include specific management restrictions). By adjusting the reference points and epsilon constraint values, new Pareto efficient solutions are possible. 

 To adjust the solver, all one needs to do is appropriately call the solver in the code. Of course, with commercial solvers appropriate permissions are required. We have currently coded a selection of solvers to be used (GLP, CPLEX, GUROBI, GLOP). To switch from one to another, we need to call the specific solver that is being used. This was hard coded into the software, however to address this comment we have incorporated a variable that can be adjusted when you call the optimization function. So now, to change solvers, you need to simply identify it when calling the function (currently it is set to a default of CLP).

 So now, when starting the GUI, the solver can be identified directly, i.e.: 

 BES = Opt.OptGUI("NFS",True,"rslt_RCP0_Bavaria_Germany_pause_2_V1.zip",solver="CLP")

 We have updated this information in the Readme.md of the software on Code Ocean, to highlight this adjustment. Only open access solvers will work on the Code Ocean system, but we have tested on a system with CPLEX, and this works nicely.

**Reviewer comments: **On the output side, the article offers three vague sentences about the program's output. I would like to know more about what is included in the output. Does it look something like Figure 3? How might the output look for a different type of forest, perhaps with different management options and different objectives? What if I want more detail about a given Pareto optimal solution? How can I visualize trade-offs among different preference sets? Figure 3 shows results for one set of preferences from the case study, but it does not illustrate how the system was used to assess trade-offs between different objectives, which, to me, is a key point of multiobjective optimization.

**Authors response: **Currently, the output of the optimization is something similar to Figure 4 (now after revisions), in addition to a table of the management options selected for each stand, with a column to indicate how much of the stand is managed according to the specific management option (as linear programming is used). This table includes all information produced by the forest simulator, so users can produce a wide range of visualizations for all variables that were simulated in the simulator (and not just those objectives and constraints that were included in the optimization).

 Yes, figure 4 does show the results of one case study, and doesn’t assess trade-offs between objectives. This wasn’t the focus of the optimization approach, nor was this the focus of the software. However, this software could be used to assess trade-offs, if desired. This would require storing a variety of solutions to enable comparisons. To visualize trade-offs between preference sets, you would need to extract solutions for each preference set and compare. Or alternatively, produce values for each objective, and compare these. If the interactive process is quick enough – users will be able to intuitively deduce trade-offs between objectives. However, from our perspective, multi-objective optimization was used to construct preference specific solutions, and not specifically to quantify and evaluate trade-offs.

 Overall, the optimization output is primarily a very large table, with the outcomes of the specific output.  A variety of graphs, maps and other visualizations are possible. A more detailed explanation we have added to manuscript, see end of section Graphical user interphase. Currently we have coded a selection of time series graphs, maps representing the spatial information of the data (if there is a corresponding geographic data). Currently we do not store the information of multiple solutions – although this is a very good idea and we will incorporate this to the next version of the software. You can store the information through a csv file, or by capturing the images constructed in the software.

**Reviewer comments: **Major comments: Constraints vs Objectives

 The article is not clear on the distinction between constraints and objectives. For example, one item listed as an objective in the section "Formulating objective functions" sounds more like a constraint than an objectives. In particular, "increase the quantity of deadwood in the forest by a specific year, and to ensure that quantity is kept for the rest of the planning horizon" sounds like a constraint to me. Furthermore, the software offers a dozen ways to create an objective but only (apparently) two ways to create a constraint. In the case study (see Table 2), objectives and constraints are mixed together with no indication which items are objectives and which are constraints.

**Authors response: **The reviewers are somewhat correct that there may be some confusion on the distinction between constraints and objectives. In this case, we are using a multi-objective optimization formulation, with a variety of functions representing the achievement of a wide variety of variables over time, evaluated as specific functions. All functions can be defined as both constraints and objectives – constraints are formulated using the epsilon constraint approach (Haimes et al. 1971) while the objectives are formulated using the achievement scalarizing function (Weizerbicki 1982) . The key difference is that the epsilon constraints are given values that are required to be above (this introduces the possibility of infeasible solutions) while objectives are given targets which the ASF strives to meet as close as possible. Thus, the clear distinction between constraints and objectives is the mathematical formulation applied to the specific function, either the epsilon constraint (at line 392) or the ASF (lines 389 and 390).

 So, when this reviewer suggests that there are “a dozen ways to create an objective”, these objectives can also be interpreted as constraints (with the difference being that a constraint is something that must be met, while the reference point objective is something that is desired). 

 The additional constraints (e.g. only CCF management on peat as in the Finnish example) are not epsilon constraints, but “hard” constraints on management. 

 Literature:

 Wierzbicki AP: On the completeness and constructiveness of parametric characterizations to vector optimization problems. OR Spektrum. 1986;8:73–87. 10.1007/BF01719738

**Reviewer comments: **Minor comments:

 The article states that: "Forest planning literature often focuses on the production of timber resources, obtaining a relative even-flow of timber over time while minimizing costs of logistics and maximizing timber production." This is not accurate. There is a large literature of forest planning models that address multiple objectives.

**Authors response: **We have change the corresponding section in the introduction (also according R1). Please see our changed section, saying that “A few centuries ago, forest planning literature often focused on the production of timber resources”. Additionally we highlighted two sentences later, that “Nowadays, forests are instead important and recognized for a wide range of societal demands” 

**Reviewer comments: **The article references two similar packages, desdeo-mcdm and optimLanduse, but it does not elaborate on what the similarities and differences are between MultiOptForest and these other packages. For example, could one use either desdeo-mcdm and optimLanduse instead of MultiOptForest?

**Authors response: **Desdeo-mcdm – is a industry independent software solutions, likely aspects could be incorporated, whereas our software is adapted to forest management planning problems. optimLanduse used optimLanduse uses an robust multi-objective land-cover composition optimization approach of Knoke et al.(2016)., while MulitOptForest used a multi-objective problem formulation ( Miettinen, 1999) in combination with the achievement scalarizing functions approach (Wierzbicki 1986). This difference we have explained now in the introduction section.

 Literature: 

 Knoke, T., Paul, C., Hildebrandt, P., Calvas, B., Castro, L.M., Hartl, F., Dollerer, M., Hamer, U., Windhorst, D., Wiersma, Y.F., Curatola Fernández, G.F., Obermeier, W.A., Adams, J., Breuer, L., Mosandl, R., Beck, E., Weber, M., Stimm, B., Haber, W., Fürst, C., Bendix, J., 2016. Compositional diversity of rehabilitated tropical lands supports multiple ecosystem services and buffers uncertainties. Nature Communications 7. https://doi.org/10.1038/ncomms11877

**Reviewer comments: **I would like to acknowledge the assistance of Silvana Nobre in developing this review.

**Authors response: **We appreciate the comments of both reviewers!

# Reviewer report: Reviewer response for version 1

**DOI:** 10.21956/openreseurope.17082.r35231

**Published:** 2023-10-06

**Authors:** Can Vatandaslar

**Affiliations:** 1 Artvin Coruh University, Artvin, Turkey

**Manuscript Type**

 Software tool article

**General comments:**

 The manuscript highlights the importance of decision support tools in multifunctional forest management planning and introduces a software, MultiOptForest, which is capable of analyzing synergies & trade-offs among different objectives and ecosystem services under various land-use scenarios. It also provides a case study to show the capability of the software.

 The software is original and it fills an important gap in management-related decision making in forestry that can be often complicated under conflicted objectives and societal demands. Although the authors state that MultiOptForest provides an interactive optimization framework to non-expert users without training in coding, I could not find a graphical user interface when I reviewed the Zenodo repository (i.e., Eyvindson et al. 2023). It is all about codes written in the Python language which can hinder the uptake of the proposed solution, especially considering the coding skills of forest managers and private landowners across the world.

 Thus, my suggestion is that the GUI of the software (as shown in the MS) should be made accessible to readers as soon as possible to speed up the uptake process of the software among decision-makers.

 My small comments related to specific sections of the MS are below:

**Specific comments:**

**Title**

 “MultiOptForest: An interactive multi-objective optimization tool for forest planning and scenario analysis”.

 The title seems clear and concise.

**Abstract**

 “… multiple forest ecosystem services and biodiversity”. According to the latest version of CICES (v5.1), biodiversity is one of the ecosystem services. So, it may be removed from the end of this sentence.

 “… impacting the development of forests…” may be revised to “…impacting the natural development of forests…” if the intent is forest growth and yield over time.

**Introduction**

 “… multiple forest ecosystem services and biodiversity, …”. Please see my previous comment for the abstract section.

 “… to plan the silvicultural and harvesting decisions…” …quantify the trade-offs between multiple forest objectives.” If MultiOptForest can also analyze the synergies among multiple ecosystem services, the authors should add this term (i.e., synergy) along with trade-offs.

 The Pareto optimal solution approach was briefly introduced in the second paragraph. Some references are needed here to inform interested readers.

 “Forest planning literature often focuses on the production of timber resources…” I do not agree with the authors. It might be somehow true before the 1990s (as shown in the work cited by the authors (Johnson & Scheurman, 1977), but today contemporary forest management plans mostly deal with multi-functionality, focusing on both economic, ecological, and sociocultural aspects of sustainability. I would suggest a broader literature search for the most recent studies across the world beyond their own work (i.e., Blattert et al. 2022).

 “…this software can be used in a variety of forest planning cases, including tactical, strategic, and operational planning cases”. How about scales? Which scale does work well with this software? For example, can it be easily run at the national scale (larger problems)? The spatial aspect and potential limitations should be stated in the manuscript. (I have noticed that the authors mentioned the scales in the discussion. A brief description would also be okay in the Intro).

**Software description**

 While incorporating stand ID, time period, and management regime into the software is clearly explained, integrating the provision of ecosystem services seems vague.

**GUI**

 I could not find the GUI in the zip file the authors provided through Zenodo.  

**Use case**

 In Table 2, Maximizing wood production is objective, while even-flow harvest is a constraint. If so, they should be separated into two cells.

**Discussion and Conclusion**

 These sections seem appropriate.

 The English grammar and academic language of the manuscript is good as a whole.

 Best regards.

Is the rationale for developing the new software tool clearly explained?

Yes

Is the description of the software tool technically sound?

Yes

Are sufficient details of the code, methods and analysis (if applicable) provided to allow replication of the software development and its use by others?

Partly

Is sufficient information provided to allow interpretation of the expected output datasets and any results generated using the tool?

Partly

Reviewer Expertise:

My areas of expertise are forest management planning, ecosystem services, and modeling. I do not have enough skills to assess the codes written in the Python language.

I confirm that I have read this submission and believe that I have an appropriate level of expertise to confirm that it is of an acceptable scientific standard.

# Response

**Published:** 2024-12-02

**Authors:** Kyle Eyvindson

**Affiliations:** MINA, Norges miljø- og biovitenskapelige universitet, Ås, Norway

**Authors response:**

 We appreciate the constructive review. To respond to the suggestions and comments, we respond to the reviewer directly.

**Reviewer comments:**

 The manuscript highlights the importance of decision support tools in multifunctional forest management planning and introduces a software, MultiOptForest, which is capable of analyzing synergies & trade-offs among different objectives and ecosystem services under various land-use scenarios. It also provides a case study to show the capability of the software. The software is original and it fills an important gap in management-related decision making in forestry that can be often complicated under conflicted objectives and societal demands. Although the authors state that MultiOptForest provides an interactive optimization framework to non-expert users without training in coding, I could not find a graphical user interface when I reviewed the Zenodo repository (i.e., Eyvindson et al. 2023). It is all about codes written in the Python language which can hinder the uptake of the proposed solution, especially considering the coding skills of forest managers and private landowners across the world.

 Thus, my suggestion is that the GUI of the software (as shown in the MS) should be made accessible to readers as soon as possible to speed up the uptake process of the software among decision-makers.

**Authors response:**

 We completely agree that the use of the GUI needs to be accessible to the users. We have tried to do this with the Code Ocean framework (https://codeocean.com/capsule/2689439/tree/v1) – as it allows for temporary (and free for academics) use of cloud computing. This allows users to test the tool without any software installations on their local computer. We now have created a step-by-step user guideline as supplementary material (but also linked to the Readme.md) allowing users to get started with the tool on how to connect with the Code Ocean framework, launching the tool, testing and running the optimization with the GUI. This should provide a good basis for accademics/forest planners. Of course, a more advanced software implementation would require additional programming efforts which is unfortunately not doable at this stage.

**Reviewer comments:**

 My small comments related to specific sections of the MS are below:

 Specific comments:

 Title

 “MultiOptForest: An interactive multi-objective optimization tool for forest planning and scenario analysis”. The title seems clear and concise.

**Authors response:**

 Thanks.

**Reviewer comments:**

 Abstract

 “… multiple forest ecosystem services and biodiversity”. According to the latest version of CICES (v5.1), biodiversity is one of the ecosystem services. So, it may be removed from the end of this sentence.

**Authors response:**

 We kindly disagree with the reviewer. Looking at the CICES (V5) classification https://cices.eu/cices-structure/ the hierarchical structure of the earlier version (CICES V4.3) has been retained in V5.1. At the highest level in the classification, services are grouped according to three Sections that relate to whether the contributions to human well-being support: 

 a) the provisioning

  of material and energy needs,

 b) regulation and maintenance of the environment for humans, or

 c) the non-material characteristics of ecosystems that affect physical and mental states of people, that is their cultural significance.

 Thus, we prefer to talk about ecosystem services “and biodiversity” in the abstract (and the manuscript), and aim to follow the classification of https://foresteurope.org/ecosystem-services/. According to this classification, biodiversity is seen as: “the variety of all life on earth – plays a key role in the structural set-up of ecosystems, which is essential to maintaining basic ecosystem processes and supporting ecosystem functions.”

**Reviewer comments:**

 “… impacting the development of forests…” may be revised to “…impacting the natural development of forests…” if the intent is forest growth and yield over time.

**Authors response:**

 We changed it to …”impacting the future development of forests”, since it can cover natural aspects as well as management aspects (planting, tending, harvesting, etc.). 

**Reviewer comments:**

 Introduction

  “… multiple forest ecosystem services and biodiversity, …”. Please see my previous comment for the abstract section.

**Authors response:**

 Please see our comment above.

**Reviewer comments:**

  “… to plan the silvicultural and harvesting decisions…” …quantify the trade-offs between multiple forest objectives.” If MultiOptForest can also analyze the synergies among multiple ecosystem services, the authors should add this term (i.e., synergy) along with trade-offs.

**Authors response:**

 We have changed it to: “This has led to a rising demand for tools to quantify the conflicts between multiple forest objectives. These tools should be able to investigate the trade-offs and synergies between objectives, …”

**Reviewer comments:**

 The Pareto optimal solution approach was briefly introduced in the second paragraph. Some references are needed here to inform interested readers.

**Authors response:**

 We have included the following references to this paragraph:

 Borges, J.G., Garcia-Gonzalo, J., Bushenkov, V., McDill, M.E., Marques, S., Oliveira, M.M., 2014. Addressing Multicriteria Forest Management With Pareto Frontier Methods: An Application in Portugal. Forest Science 60, 63-72. http://dx.doi.org/10.5849/forsci.12-100 

 Díaz-Yáñez, O., Pukkala, T., Packalen, P., Lexer, M.J., Peltola, H., 2020. Multi-objective forestry increases the production of ecosystem services. Forestry: An International Journal of Forest Research. https://doi.org/10.1093/forestry/cpaa041 

 Mazziotta, A., Podkopaev, D., Triviño, M., Miettinen, K., Pohjanmies, T., Mönkkönen, M., 2017. Quantifying and resolving conservation conflicts in forest landscapes via multiobjective optimization. https://www.silvafennica.fi/article/1778 

**Reviewer comments:**

 “Forest planning literature often focuses on the production of timber resources…” I do not agree with the authors. It might be somehow true before the 1990s (as shown in the work cited by the authors (Johnson & Scheurman, 1977), but today contemporary forest management plans mostly deal with multi-functionality, focusing on both economic, ecological, and sociocultural aspects of sustainability. I would suggest a broader literature search for the most recent studies across the world beyond their own work (i.e., Blattert et al. 2022).

**Authors response:**

 Thanks for the comment. We have re-phrased the corresponding section; highlighting, that “A few centuries ago, forest planning literature often focused on the production of timber” (see paragraph three).

 We also highlighted that “Nowadays, forest are instead important and recognized for a wide range of societal demands (LIT-1), why there is a need to shift towards a flexible approach accounting for those multi-objectives (LIT-2).” Those statement we complemented by following Literature

 LIT-1:

 MEA, 2005. Millenium Ecosystem Assessment - Ecosystem and Human Well-being: Snthesis. Island Press, Washington, DC.

 Pascual, U., Balvanera, P., Díaz, S., Pataki, G., Roth, E., Stenseke, M., Watson, R.T., Başak Dessane, E., Islar, M., Kelemen, E., Maris, V., Quaas, M., Subramanian, S.M., Wittmer, H., Adlan, A., Ahn, S., Al-Hafedh, Y.S., Amankwah, E., Asah, S.T., Berry, P., Bilgin, A., Breslow, S.J., Bullock, C., Cáceres, D., Daly-Hassen, H., Figueroa, E., Golden, C.D., Gómez-Baggethun, E., González-Jiménez, D., Houdet, J., Keune, H., Kumar, R., Ma, K., May, P.H., Mead, A., O’Farrell, P., Pandit, R., Pengue, W., Pichis-Madruga, R., Popa, F., Preston, S., Pacheco-Balanza, D., Saarikoski, H., Strassburg, B.B., van den Belt, M., Verma, M., Wickson, F., Yagi, N., 2017. Valuing nature’s contributions to people: the IPBES approach. Current Opinion in Environmental Sustainability 26-27, 7-16. https://doi.org/10.1016/j.cosust.2016.12.006

 Winkel, G., Lovrić, M., Muys, B., Katila, P., Lundhede, T., Pecurul, M., Pettenella, D., Pipart, N., Plieninger, T., Prokofieva, I., Parra, C., Pülzl, H., Roitsch, D., Roux, J.-L., Thorsen, B.J., Tyrväinen, L., Torralba, M., Vacik, H., Weiss, G., Wunder, S., 2022. Governing Europe's forests for multiple ecosystem services: Opportunities, challenges, and policy options. Forest Policy and Economics 145, 102849. https://doi.org/10.1016/j.forpol.2022.102849 

 LIT-2:

 Augustynczik, A.L.D., Gutsch, M., Basile, M., Suckow, F., Lasch, P., Yousefpour, R., Hanewinkel, M., 2020. Socially optimal forest management and biodiversity conservation in temperate forests under climate change. Ecological Economics 169, 106504. https://doi.org/10.1016/j.ecolecon.2019.106504¨

 Eggers, J., Lundström, J., Snäll, T., Öhman, K., 2022. Balancing wood production and biodiversity in intensively managed boreal forest. Scandinavian Journal of Forest Research 37, 213-225. https://doi.org/10.1080/02827581.2022.2066170

 Uhde, B., Heinrichs, S., Stiehl, C.R., Ammer, C., Müller-Using, B., Knoke, T., 2017. Bringing ecosystem services into forest planning – Can we optimize the composition of Chilean forests based on expert knowledge? Forest Ecology and Management 404, 126-140. https://doi.org/10.1016/j.foreco.2017.08.021

**Reviewer comments:**

 “…this software can be used in a variety of forest planning cases, including tactical, strategic, and operational planning cases”. How about scales? Which scale does work well with this software? For example, can it be easily run at the national scale (larger problems)? The spatial aspect and potential limitations should be stated in the manuscript. (I have noticed that the authors mentioned the scales in the discussion. A brief description would also be okay in the Intro).

**Authors response:**

 We appreciate this comment, the issue of scale can be related to the time taken in the data processing and optimization. We have applied the software with several very large problems and were able to manage successfully. As the reviewer noted, the scale aspect in the discussion (second last paragraph). However, we briefly specified the flexibility of the tool (applicable to large and small scale) also at the end of the intro saying: “As a flexible approach to constructing multi-objective optimization formulations, this software can be used in a variety of forest planning cases, including large scale strategic planning cases (e.g. national forest inventory plot level) as well as small scale tactical and operational planning cases (e.g. individual forest stands of a management unit).”

 The software works well for both scales. However, computation time and complexity depend on the number of the number of individual optimized entities (forests plots or stands), which we have added to the discussion.

**Reviewer comments:**

 Software description

 While incorporating stand ID, time period, and management regime into the software is clearly explained, integrating the provision of ecosystem services seems vague.

**Authors response:**

 We improved the description of indexed input data to the provided ecosystem services and biodiversity and wrote: “… columns for indicators represent the state of the forest and of the ecosystem service and biodiversity. Those indicators are simulated for each entity (forest stand or plot level) under alternative managements.”

**Reviewer comments:**

 GUI

 I could not find the GUI in the zip file the authors provided through Zenodo. 

**Authors response:**

 The GUI is not found on the Zenodo, the Zenodo is only a repository of the python code (as the reviewer has correctly noted). To enable readers to interact with the code, we have loaded the code as a project in the virtual environment on Code Ocean, where those interested can test and explore the approach. https://codeocean.com/capsule/2689439/tree/v1 . 

 We generated a step-by-step guide illustrating with screen shots how the tool can virtually launched via CodeOcean, providing as such an illustrative example how user can get familiar with the tool and explore pre-defined cases/optimization problems, including: how to start the tool on Code Ocean (start editing), how to Launch Jupiter notebook, selecting one of the country example, and run the Jupyter Notebook, which loads the GUI where constraints/objective can be set.

 The step-by-step user guide was further included in the ReadMe file, showing now two sub-section under “Getting started”: Online on Code Ocean, and “On local system”

**Reviewer comments:**

 Use case

 In Table 2, Maximizing wood production is objective, while even-flow harvest is a constraint. If so, they should be separated into two cells.

**Authors response:**

 We highlighted, which aspects are addressed as objectives, and which ones as constraints. Wood production and Bioenergy where implemented as objective that maximize the minimum indicator values (harvested roundwood or biomass) over all simulated years, which results in an even-flow of wood or biomass production. This we have further explained in the text and table 2.

**Reviewer comments:**

 Discussion and Conclusion

 These sections seem appropriate.

**Authors response:**

 Thanks

**Reviewer comments:**

 The English grammar and academic language of the manuscript is good as a whole.

**Authors response:**

 Thanks
